# Structure of the mouse acidic amino acid decarboxylase GADL1

**DOI:** 10.1107/S2053230X17017848

**Published:** 2018-01-01

**Authors:** Arne Raasakka, Elaheh Mahootchi, Ingeborg Winge, Weisha Luan, Petri Kursula, Jan Haavik

**Affiliations:** a Department of Biomedicine, University of Bergen, Jonas Lies Vei 91, 5009 Bergen, Norway; b K. G. Jebsen Centre for Research on Neuropsychiatric Disorders, University of Bergen, Jonas Lies Vei 91, 5009 Bergen, Norway; c Faculty of Biochemistry and Molecular Medicine, University of Oulu, PO Box 5400, 90014 Oulu, Finland; d Division of Psychiatry, Haukeland University Hospital, Bergen, Norway

**Keywords:** decarboxylases, pyridoxal phosphate, catalysis, conformational change

## Abstract

Pyridoxal 5′-phosphate (PLP) is a ubiquitous cofactor in various enzyme classes, including PLP-dependent decarboxylases. A recently discovered member of this class is glutamic acid decarboxylase-like protein 1 (GADL1), which lacks the activity to decarboxylate glutamate to γ-aminobutyrate, despite its homology to glutamic acid decarboxylase. Among the acidic amino acid decarboxylases, GADL1 is most similar to cysteine sulfinic acid decarboxylase (CSAD), but the physiological function of GADL1 is unclear, although its expression pattern and activity suggest a role in neurotransmitter and neuroprotectant metabolism. The crystal structure of mouse GADL1 is described, together with a solution model based on small-angle X-ray scattering data. While the overall fold and the conformation of the bound PLP are similar to those in other PLP-dependent decarboxylases, GADL1 adopts a more loose conformation in solution, which might have functional relevance in ligand binding and catalysis. The structural data raise new questions about the compactness, flexibility and conformational dynamics of PLP-dependent decarboxylases, including GADL1.

## Abstract 2

The structure of the mouse glutamic acid decarboxylase-like protein 1 (GADL1) is described. The structure provides new insights into the function of GADL1 and related decarboxylases.

## 1. Introduction   

Pyridoxal 5′-phosphate (PLP), or vitamin B_6_, is a versatile cofactor that is involved in many enzymatic reactions spanning multiple enzyme classes and chemical reactions (Percudani & Peracchi, 2003 ▸). PLP-dependent decarboxylases constitute a large family of enzymes that catalyze a range of metabolic reactions. Many of these enzymes catalyze biologically well defined processes; inactivating mutations affecting them are associated with severe phenotypes, and some of them are treatment targets in human disease (Baekkeskov *et al.*, 1990 ▸; Eliot & Kirsch, 2004 ▸; El-Sayed & Shindia, 2011 ▸; Paiardini *et al.*, 2014 ▸; Sköldberg *et al.*, 2004 ▸).

Despite extensive research, the biological functions of many PLP-dependent enzymes are still unclear. One such enzyme is glutamic acid decarboxylase-like protein 1 (GADL1), which was originally named based on its sequence homology to glutamic acid decarboxylase (GAD), which synthesizes the inhibitory neurotransmitter γ-aminobutyric acid (GABA; Fenalti *et al.*, 2007 ▸). However, GADL1 has no reactivity towards glutamic acid and therefore is unlikely to be involved in GABA signalling (Liu *et al.*, 2012 ▸; Winge *et al.*, 2015 ▸). It has been suggested that GADL1 is involved in taurine production (Liu *et al.*, 2012 ▸). On the other hand, in our recent comparative study of GADL1 and cysteine sulfinic acid decarboxylase (CSAD), an enzyme homologous to GADL1 that is involved in taurine biosynthesis, we showed that these enzymes act differently. Compared with CSAD, the activity of GADL1 towards cysteine sulfinic acid (CSA) as a substrate is much lower, and GADL1 has a stronger preference for aspartate, suggesting that GADL1 may be involved in the biosynthesis of β-alanine and its peptide derivatives, such as the neuro­protectant carnosine (Min *et al.*, 2008 ▸; Park *et al.*, 2014 ▸; Winge *et al.*, 2015 ▸).

We showed in our earlier study that mouse and human brain have distinct patterns of expression of CSAD and GADL1 (Winge *et al.*, 2015 ▸). Whereas both CSAD and GADL1 were expressed in neurons, only the CSAD mRNA was detected in astrocytes. Using a homology model of GADL1 based on the crystal structure of human CSAD (*Hs*CSAD), we performed *in silico* screening of potential substrate analogues and discovered the first generation of inhibitors with partial selectivity against either GADL1 or CSAD. However, detailed mechanistic studies have been hampered by the lack of structural information.

In this study, we describe the crystal structure of mouse GADL1 (*Mm*GADL1). Additionally, we used small-angle X-ray scattering (SAXS) to elucidate the solution shape of *Mm*GADL1. The overall fold of *Mm*GADL1 is similar to those of CSAD and other close homologues, with a flexible loop, not defined in electron density, from the apposing monomer covering the active site, which is possibly relevant in catalysis. SAXS data demonstrate that *Mm*GADL1 adopts a loosened state in solution, which might correspond to an open conformation significant for cofactor or substrate binding.

## 2. Materials and methods   

### 2.1. Macromolecule production   

#### 2.1.1. Construct preparation, protein expression and purification   

The expression vector for *Mm*GADL1 was prepared in the Gateway system using pTH27 (Hammarström *et al.*, 2006 ▸) as the destination vector. Cloning involved a two-step PCR protocol and homologous recombination into Gateway vectors using standard procedures. The resulting construct codes for an N-terminal His_6_ tag, a *Tobacco etch virus* (TEV) protease cleavage site and *Mm*GADL1 residues 1–502 (UniProt entry E9QP13). A longer isoform of *Mm*GADL1 also exists (UniProtKB entry Q80WP8), and the construct corresponds to residues 49–550 of this isoform.

#### 2.1.2. Expression and purification of *Mm*GADL1   

His_6_-tagged *Mm*GADL1 was expressed in *Escherichia coli* BL21-CodonPlus(DE3)-RIPL cells (Stratagene) at 288 K using 150 m*M* IPTG induction. Cell pellets were lysed by sonication in a buffer consisting of 50 m*M* sodium phosphate buffer pH 7.4, 500 m*M* NaCl, 20 m*M* imidazole, 0.2 mg ml^−1^ lysozyme, 1 m*M* MgCl_2_, 2 m*M* pyridoxine hydrochloride and cOmplete EDTA-free protease inhibitors (Roche). Phenylmethyl­sulfonyl fluoride was added to 1 m*M* immediately following sonication. The unclarified lysate was applied directly onto an IMAC HiTrap TALON crude column (GE Healthcare). The column was washed with 50 m*M* sodium phosphate pH 7.4, 500 m*M* NaCl, 50 m*M* sodium phosphate pH 7.4, 500 m*M* NaCl, 20 m*M* imidazole. Elution was carried out with 100 m*M* imidazole in 50 m*M* sodium phosphate pH 7.4, 500 m*M* NaCl. Size-exclusion chromatography was performed using a Superdex HR 200 column (GE Healthcare) equilibrated with 20 m*M* HEPES, 200 m*M* NaCl pH 7.5.

### 2.2. Crystallization   

Initial crystallization conditions for *Mm*GADL1 were obtained from the JCSG-*plus* screen (Molecular Dimensions) using sitting-drop vapour diffusion. The crystallization conditions, which yielded crystals with a needle morphology arranged as single crystals or point-originated clusters, were comprised of 80 m*M* sodium cacodylate pH 6.0–7.4, 13–14%(*w*/*v*) PEG 8000, 120–160 m*M* calcium acetate, 15.0–17.5%(*w*/*v*) glycerol. 0.3–1.5 µl drops with different volume ratios of protein solution (6.5–7.5 mg ml^−1^ in 20 m*M* HEPES pH 7.4, 200 m*M* NaCl) and reservoir solution were used at 281 and 293 K, equilibrating against 40 µl reservoir solution. Crystals were briefly soaked in cryoprotectant solutions and flash-cooled in liquid N_2_. The detailed conditions used to obtain the crystals used for data collection are given in Table 1 ▸.

### 2.3. Data collection, structure solution and refinement   

The *Mm*GADL1 structure was solved from the two crystal forms by molecular replacement in *Phaser* (McCoy *et al.*, 2007 ▸) using the structure of *Hs*CSAD (PDB entry 2jis; Structural Genomics Consortium, unpublished work) as the search model. Crystal form 1 diffracted to 3 Å resolution, while crystal form 2, which was used for initial structure solution, diffracted to 2.4 Å resolution; the latter suffered from near-perfect pseudomerohedral twinning and high translational pseudosymmetry. Owing to these observations, both crystal forms were solved and initially refined, and the lower resolution structure, which completely lacked twinning and pseudotranslation, was considered to be better for final refinement. In essence, despite the higher nominal resolution, the twinned crystal suffering from pseudotranslation gave lower-quality electron-density maps. The twinning and pseudotranslation operations are given in Table 2 ▸. NCS restraints were employed throughout refinement. Refinement was performed with *phenix.refine* (Afonine *et al.*, 2012 ▸) and model building with *Coot* (Emsley & Cowtan, 2004 ▸). The structure was validated with *MolProbity* (Chen *et al.*, 2010 ▸). Data collection and refinement statistics can be found in Table 2 ▸. The resolution limits used were based on recent studies (Karplus & Diederichs, 2015 ▸) showing that useful information is available for refinement even for data with a CC_1/2_ much lower than 50%.

### 2.4. Small-angle X-ray scattering   

Synchrotron SAXS data were collected on the EMBL/DESY BioSAXS beamline P12 (Blanchet *et al.*, 2015 ▸). The protein was at 1.6–6.5 mg ml^−1^ in 20 m*M* HEPES pH 7.4, 200 m*M* NaCl. The scattering data were processed and analyzed with programs from the *ATSAS* package (Konarev *et al.*, 2006 ▸). The molecular weight was determined by comparison of the forward scattering intensity, *I*(0), with a fresh monomeric bovine serum albumin standard. Models of *Mm*GADL1 were built with *GASBOR* (Svergun *et al.*, 2001 ▸) and *SREFLEX* (Panjkovich & Svergun, 2016 ▸), using data extrapolated to zero concentration. Theoretical scattering curves from crystal structure coordinates were calculated with *CRYSOL* (Svergun *et al.*, 1995 ▸). Model superposition was performed using *SUPCOMB* (Kozin & Svergun, 2001 ▸). Details of SAXS data collection, processing and analysis are given in Table 3 ▸, and the raw SAXS scattering data are provided as Supporting Information.

### 2.5. Sequence and structure analysis   

*APBS* (Unni *et al.*, 2011 ▸), *UCSF Chimera* (Pettersen *et al.*, 2004 ▸), *PyMOL* (http://www.pymol.org), *ProtParam* (Gasteiger *et al.*, 2005 ▸), *PDBeFold* (Krissinel & Henrick, 2004 ▸), *MUSCLE* (Edgar, 2004 ▸) and *ESPript*3.0 (Robert & Gouet, 2014 ▸) were used for bioinformatics and structure analyses.

## 3. Results and discussion   

### 3.1. The crystal structure of *Mm*GADL1   

Initial screening for crystallization conditions of *Mm*GADL1 resulted in a single condition that produced diffracting crystals. Crystals formed with a needle morphology, often growing in bunches or clusters and generally being very thin, with maximum lengths reaching 500 µm. The diffraction quality was initially poor, with diffraction limits of around 6–7 Å and highly smeared reflections. By optimizing the size, the amount of nucleation, and the general appearance of the crystals, the conditions given in Table 1 ▸ produced thin but nonfragile crystals that allowed structure refinement in two crystal forms to resolutions of 2.4 and 3.0 Å; the higher resolution data set was plagued by significant twinning and pseudotranslation (Table 2 ▸). Thus, the structure of the nontwinned crystal form is discussed here; essentially all features can also be seen in the twinned crystal.

**Figure 1:**
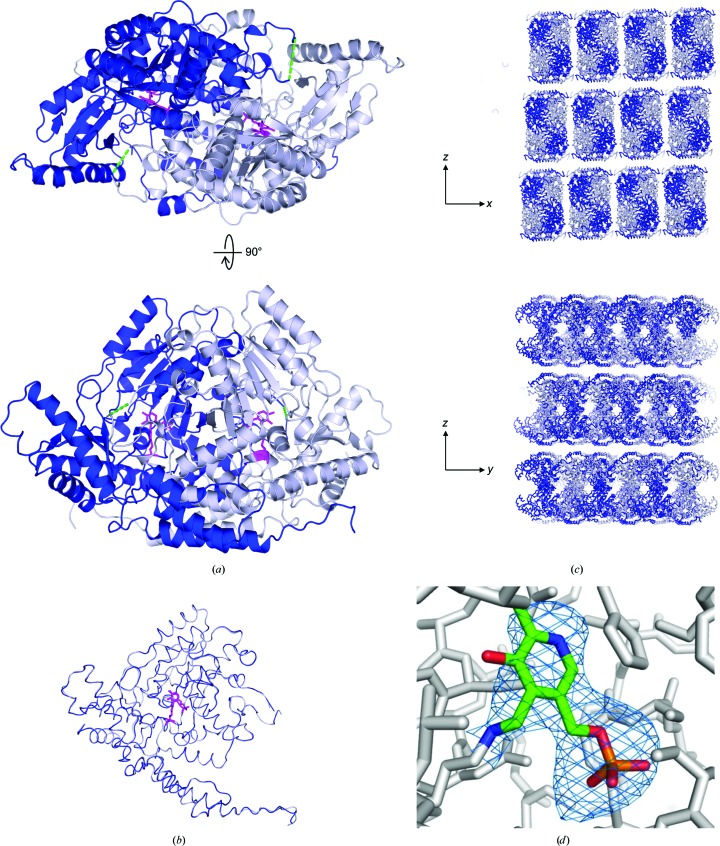
Overall structure of *Mm*GADL1. (*a*) The *Mm*GADL1 dimer. The PLP molecule covalently bound to Lys314 is shown in magenta. The green dashed line indicates the position of the flexible loop covering the active site. (*b*) Superposition of the two *Mm*GADL1 monomers in the homodimer. The covalently bound PLP is shown in magenta. The two monomers are essentially identical. (*c*) Crystal lattice arrangement of *Mm*GADL1 in two different planes. Note the uniform 11 Å cavities in the *xy* plane between protein layers. (*d*) Omit *F*_o_ − *F*_c_ difference map (blue mesh) contoured at 2σ for the covalently bound PLP cofactor on Lys314.

The crystal structure revealed a single *Mm*GADL1 homodimer in the asymmetric unit, which was the expected oligomeric state based on other PLP-dependent decarboxylases (Fig. 1 ▸ *a*). For both chains, residues 11–502 could be built, with the flexible loop common to PLP-dependent decarboxylases (Fenalti *et al.*, 2007 ▸) being absent from the electron density (approximately residues 340–350). The overall conformation of the two chains was nearly identical (Fig. 1 ▸ *b*).

In the crystal lattice, the protein dimers form layers in the *xy* plane, separated by a rather uniform spacing (Fig. 1 ▸ *c*). As the first ∼30 amino acids of the tagged protein were not visible, they are most likely to occupy the space between protein dimers in the crystal. This is the likely source of the high mosaicity and incomplete lattice arrangement in the current *Mm*GADL1 crystals.

**Figure 2:**
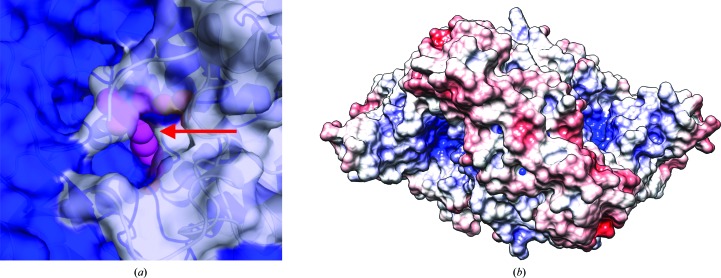
The *Mm*GADL1 active site. (*a*) Close-up view of the active-site cavity with the reactive moiety of PLP (magenta) visible (red arrow). Note how the cofactor lies at the interface between two protein monomers (blue and grey). (*b*) Surface electrostatics of *Mm*GADL1. The positively charged cavity corresponds to the active site (blue).

Both active sites in the dimer are occupied by the PLP cofactor, which is covalently bound to the N^∊^ atom of Lys314 in each chain through a Schiff base linkage, being located at the dimer interface (Fig. 1 ▸ *d*). Closer observation of the active-site cavity reveals that only the active imine of the linked PLP is solvent-exposed, and it resides at the end of a narrow cavity, which represents the substrate-binding pocket (Fig. 2 ▸ *a*). Electrostatic surface analysis reveals the GADL1 active site to have a high positive charge potential (Fig. 2 ▸ *b*). This is logical, as the substrates of GADL1 are acidic amino acids; the basic nature of the binding cavity is involved in electrostatic interactions that attract and bind the substrate, orienting it correctly for catalysis.

In our earlier work, we showed that GADL1 can use aspartate and CSA as substrates, but not glutamate (Winge *et al.*, 2015 ▸). While the catalytic cavities of GAD and GADL1 are very similar, it is currently difficult to pinpoint which features of the active site might be responsible for selectivity between such similar substrates. High-resolution structures of GADL1 and its closest homologues with bound active-site ligands will clearly be required. Importantly, a large part of the active-site cavity wall will be formed by the flexible loop in the substrate-bound state; the flexible loop is invisible in most PLP-dependent decarboxylase crystal structures, but harbours a Tyr residue that is likely to be important for catalysis.

### 3.2. *Mm*GADL1 adopts an open conformation in solution   

**Figure 3:**
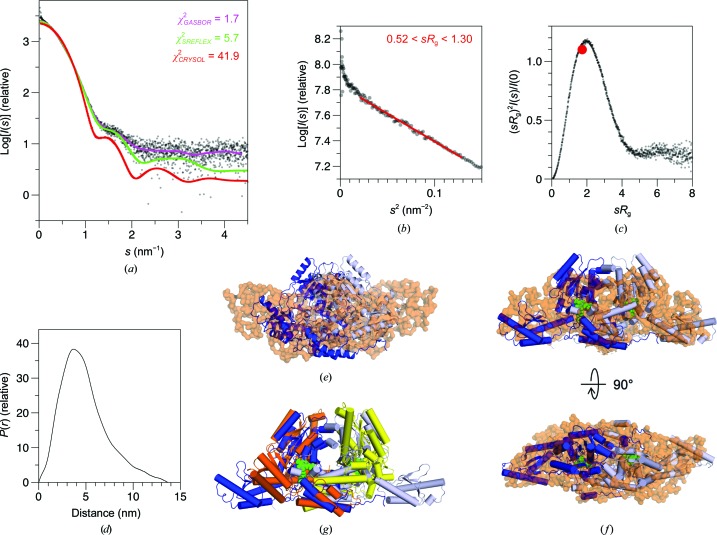
Structure of *Mm*GADL1 in solution determined by SAXS. (*a*) Raw SAXS data (dots) overlaid with *GASBOR* (pink) and *SREFLEX* (green) model fits, as well as the theoretical scattering curve calculated from the crystal structure using *CRYSOL* (red). (*b*) Guinier plot. (*c*) The dimensionless Kratky plot indicates that *Mm*GADL1 is folded, with limited flexibility. The red dot indicates the theoretical position of the peak in a folded globular protein. (*d*) Distance distribution of *Mm*GADL1. (*e*) The *GASBOR* model (orange) superimposed with the crystal structure of *Mm*GADL1 indicates a much more elongated conformation in solution. (*f*) Superposition of the *SREFLEX* (blue/grey) and *GASBOR* (orange) models suggests conformational changes relative to the crystal structure. (*g*) Comparison of the ‘open’ conformation of DDC (orange/yellow; Giardina *et al.*, 2011 ▸) and the open conformation of *Mm*GADL1 (blue/grey) seen here in solution.

We used SAXS to validate the crystal structure and to determine the conformation of *Mm*GADL1 in solution (Fig. 3 ▸, Table 3 ▸). As is apparent from the scattering data and the dimensionless Kratky plot, GADL1 presents a folded shape. While the molecular mass calculated from the forward scattering intensity accurately matches that of a dimer, the theoretical scattering curve calculated from the crystal structure differs significantly. The shape in solution is more elongated than in the crystal. The radii of gyration between the theoretical scattering curve from the crystal structure and the experimental value from Guinier analysis differ by nearly 1 nm, indicating a large difference in conformation. The maximum diameter is 3 nm larger in solution than in the crystal state.

The *GASBOR* chain-like dummy residue model of *Mm*GADL1 is elongated compared with the crystal structure (Fig. 3 ▸ *e*). Attempts to model the missing N-terminal portion, while keeping the dimeric crystal structure fixed, did not fit the experimental SAXS data well (data not shown). We thus employed the recently described *SREFLEX* method (Panjkovich & Svergun, 2016 ▸) to take advantage of normal-mode analysis of the crystal structure in SAXS modelling. The *SREFLEX* model fits the scattering data well and suggests a clearly loosened solution conformation (Fig. 3 ▸ *f*), in contrast to the compact globular structures observed for PLP-dependent decarboxylases in the crystalline state. The open conformation is not similar to the open conformation observed for DOPA decarboxylase in the crystalline state (Giardina *et al.*, 2011 ▸; Fig. 3 ▸ *g*), in which case the opening occurs in the centre of the dimer. The observed open–close movement is likely to be of functional relevance in the GADL1 catalytic cycle. Whether the different conformations are linked to the binding of ligands remains to be studied. While our GADL1 preparation is enzymatically active (Winge *et al.*, 2015 ▸), and the crystal is apparently fully occupied with covalently bound PLP, we cannot currently rule out the presence of PLP-deficient GADL1 in the purified SAXS sample, since crystallization might have enriched a cofactor-bound conformation of the protein.

### 3.3. Comparison to other PLP-dependent decarboxylases   

**Figure 4:**
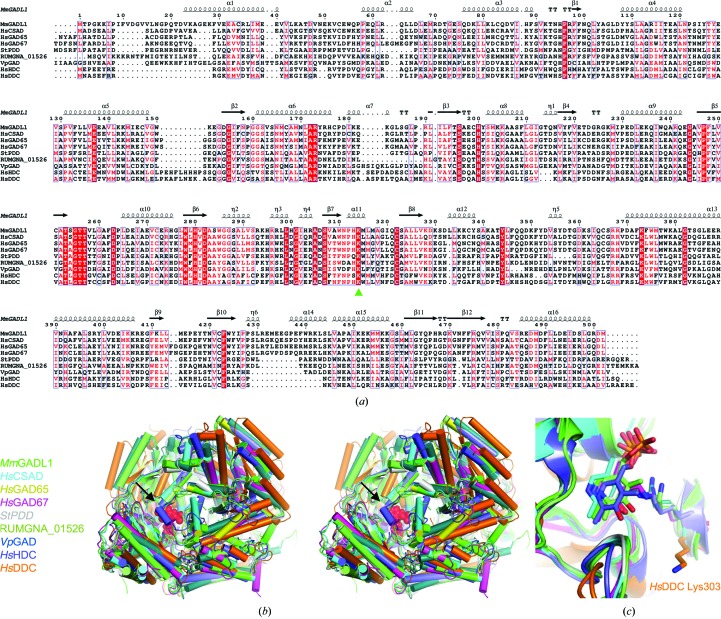
Comparison of *Mm*GADL1 with other PLP-dependent decarboxylases. (*a*) Sequence alignment of *Mm*GADL1 with homologous structures. The conserved PLP-modified lysine is indicated (green triangle). Secondary structure elements and sequence numbering correspond to *Mm*GADL1. (*b*) Stereo image of a structural superposition of PLP-dependent decarboxylase homologues, viewed towards the active-site cavity. The covalently linked *Mm*GADL1 PLP moiety is depicted as red spheres. The black arrow shows the position of the active-site-covering loop, which is disordered in the *Mm*GADL1 crystal structure. (*c*) Conservation of PLP conformation in the superposed PLP-dependent decarboxylase structures.

While *Mm*GADL1 and its homologues show low sequence conservation, apart from a few fully conserved core elements around the active site (Fig. 4 ▸ *a*), comparison of the structures of *Mm*GADL1 and its homologues reveals a conserved structural fold with little variance in the arrangement of the PLP-linked Lys residue (Figs. 4 ▸ *b* and 4 ▸ *c*, Table 4 ▸). The sequence conservation between *Mm*GADL1, *Hs*CSAD and *Hs*GADs (Fenalti *et al.*, 2007 ▸) is higher than those with human histidine decarboxylase (HDC; Komori *et al.*, 2012 ▸) and DOPA decarboxylase (DDC; Giardina *et al.*, 2011 ▸; Winge *et al.*, 2015 ▸). The latter present similar levels of sequence homology to GADL1 as the bacterial enzymes *Sphaerobacter thermophilus* PLP-dependent decarboxylase (StPDD), *Vibrio parahaemolyticus* GAD (VpGAD) and the tryptamine-synthesizing enzyme from the gut bacterium *Ruminococcus gnavus* (RUMGNA_01526; Williams *et al.*, 2014 ▸). Despite low sequence homology, the *R. gnavus* enzyme displays high structural similarity to GADL1, suggesting conservation of the fold of PLP-dependent decarboxylases involved in neurotransmitter synthesis. It is interesting to note that the absence of PLP in the active site neither alters the overall tightness of the superposed proteins nor changes the position of the conserved Lys in most structures. In the future, experimental solution-state methods, such as SAXS, may help to distinguish functionally relevant conformational states from possible crystallographic artifacts. These observations conflict with earlier results described for *Hs*DDC in the beginning of its PLP accumulation-dependent conformational landscape, where a more open conformation was evident in the crystalline state with the active-site Lys residue oriented away from the PLP-binding pocket (Giardina *et al.*, 2011 ▸).

The substrate specificity and physiological function of GADL1 remain enigmatic. However, the conserved structural details and distinct expression patterns of GADL1 suggest that it plays a specific physiological role. Variants of GADL1 have been linked to lithium response in bipolar disorder patients (Chen *et al.*, 2014 ▸), suggesting a role for GADL1 in lithium pharmacodynamics or kinetics. However, these findings have not been replicated by others, and they have been subject to much controversy (Birnbaum *et al.*, 2014 ▸; Cruceanu *et al.*, 2015 ▸; Kotambail *et al.*, 2015 ▸; Winge *et al.*, 2015 ▸; Chen *et al.*, 2016 ▸).

Owing to their common mechanistic features, many PLP-dependent enzymes are able to catalyze multiple biochemical reactions, making it difficult to define their primary biological function (Percudani & Peracchi, 2003 ▸). Of the known GADL1 substrates, Asp appears as the most characteristic substrate for GADL1 (Winge *et al.*, 2015 ▸), although the *K*_m_ is relatively high and the catalytic efficiency is low. Nevertheless, the *K*_m_ of GADL1 for Asp is in the physiological range, and one could speculate that GADL1 is involved in sensing selected metabolite levels. Relatively low binding affinity is a hallmark of many proteins with signalling roles, which have evolved as sensors of ligand availability; such proteins include, for example, the calcium sensors calmodulin and annexin (Kursula, 2014 ▸; Monastyrskaya *et al.*, 2007 ▸). Conformational flexibility, as observed here for GADL1 in solution, might have relevance in such a function.

## 4. Concluding remarks   

The physiological functions and enzymatic properties of GADL1 are subject to further studies. The structure of *Mm*GADL1 and its flexibility in solution, coupled to structural conservation with other PLP-dependent enzymes, point towards functional relevance of these features within the enzyme family. Important future work will concentrate on high-resolution structural details of substrate and inhibitor binding by GADL1, and on comparing these with those of CSAD, GAD and other PLP-dependent decarboxylases. Crucial topics to address will include the fine details of substrate specificity determinants in PLP-dependent decarboxylases, as well as the relevance of the conformational changes observed here to the catalytic cycle of this family of enzymes.

## Supplementary Material

PDB reference: mouse GADL1, 6enz

ASCII file representing the background-corrected SAXS scattering profile, extrapolated to zero concentration, with errors.. DOI: 10.1107/S2053230X17017848/tb5122sup1.txt

Supplementary Figure 1. Dependence of Rg and I(0) on sample concentration in SAXS.. DOI: 10.1107/S2053230X17017848/tb5122sup2.pdf

Raw SAXS scattering data with all concentrations.. DOI: 10.1107/S2053230X17017848/tb5122sup3.zip

## Figures and Tables

**Table 1:** Crystallization

Crystal form	1	2
Method	Sitting-drop vapour diffusion	Sitting-drop vapour diffusion
Plate type	Swissci 3-drop 96-well plate	Swissci 3-drop 96-well plate
Temperature (K)	293	281
Protein concentration (mg ml^−1^)	7.5	7
Buffer composition of protein solution	20 m*M* HEPES pH 7.4, 200 m*M* NaCl	20 m*M* HEPES pH 7.4, 200 m*M* NaCl
Composition of reservoir solution	80 m*M* sodium cacodylate pH 6.0, 14% PEG 8000, 160 m*M* calcium acetate, 15% glycerol	80 m*M* sodium cacodylate pH 7.0, 13% PEG 8000, 160 m*M* calcium acetate, 15% glycerol
Volume and ratio (protein:well solution) of drop	0.3 µl (1:1)	1.5 µl (1:2)
Volume of reservoir (µl)	40	40
Cryoprotection solution	75%(*v*/*v*) reservoir + 25%(*v*/*v*) PEG 200	80%(*v*/*v*) reservoir + 20%(*v*/*v*) glycerol

**Table 2:** Data collection, processing and structure refinement Values in parentheses are for the highest resolution shell.

Crystal form	1	2
Data-collection statistics		
Wavelength (Å)	1.0332	0.9763
Space group	*C*2	*P*2_1_
*a*, *b*, *c* (Å)	137.4, 80.6, 128.5	80.9, 121.7, 101.1
α, β, γ (°)	90, 117.9, 90	90, 90.08, 90
Resolution range (Å)	50–3.00 (3.08–3.00)	50–2.40 (2.46–2.40)
Completeness (%)	99.4 (99.5)	98.7 (95.9)
Multiplicity	6.4 (6.0)	3.6 (3.1)
〈*I*/σ(*I*)〉†	7.1 (0.7)	5.9 (0.9)
*R*_meas_	0.328 (3.535)	0.188 (1.466)
*R*_p.i.m._	0.130 (1.443)	0.099 (0.832)
CC_1/2_ (%)	98.9 (28.4)	99.3 (61.4)
Overall *B* factor from Wilson plot (Å^2^)	67	44
Refinement statistics		
Resolution range (Å)	50–3.0	—
Final *R*_cryst_	0.236	—
Final *R*_free_	0.288	—
R.m.s.d.s		
Bond lengths (Å)	0.003	—
Bond angles (°)	0.7	—
Average *B* factor (Å^2^)	91.0	—
Ramachandran plot		
Favoured (%)	92.0	—
Outliers (%)	0.6	—
*MolProbity* score [percentile]	1.98 [99th]	—
Twin operator/twin fraction (%)	—	*h*, −*k*, −*l*/45
Pseudotranslation operator/fraction (%)	—	0.060, −0.500, 0.417/38

† The mean *I*/σ(*I*) in the outermost shell falls below 2.0 at 3.3 Å for crystal form 1 and 2.7 Å for crystal form 2.

**Table 3:** Small-angle X-ray scattering

Data-collection parameters	
Protein	*Mm*GADL1, His-tagged
Instrument	P12, PETRA III, EMBL/DESY with Dectris PILATUS 2M detector (Blanchet *et al.*, 2015 ▸)
Wavelength (nm)	0.124
Beam size (µm)	200 × 120
Detector distance (m)	3.1
Angular range (nm^−1^)	0.018–4.607
Exposure time per frame (s)	0.045
No. of frames per sample	20
Monitoring for radiation damage	Data frame-by-frame comparison
Scaling method	Buffer-subtracted data normalized to 1 mg ml^−1^
Normalization	To transmitted intensity by beamstop counter (Blanchet *et al.*, 2015 ▸)
Concentration range (mg ml^−1^)	1.6, 3.3, 6.5
Temperature (K)	293
Structural parameters	
*R*_g_ from crystal structure (nm)	2.95
*D*_max_ from crystal structure (nm)	10.67
Guinier analysis	
*I*(0) from Guinier (relative)	2519
*R*_g_ from Guinier (nm)	3.62
*s*_min_ (nm^−1^)	0.143
*sR*_g_ range	0.52–1.30
Fidelity	0.92
*p*(*r*) analysis	
*I*(0) from *p*(*r*) (relative)	2557
*R*_g_ from *p*(*r*) (nm)	3.80
*D*_max_ (nm)	13.67
*s* range (nm^−1^)	0.143–1.98
Quality of fit (total estimate from *GNOM*)	0.83
Porod volume (nm^3^) (ratio to calculated molecular mass in kDa)	196 (1.6)
Molecular-mass determination	
Molecular mass from *I*(0) using *p*(*r*) (kDa) (ratio to theoretical monomer)	121.0 (2.0)
Molecular mass from *I*(0) using Guinier (kDa) (ratio to theoretical monomer)	120.6 (2.0)
Theoretical monomeric molecular mass from sequence (kDa)	60.4
Shape and atomistic modelling	
*CRYSOL* (comparison to crystal structure)	
χ^2^ value *versus* crystal structure	41.9
*s* range (nm^−1^)	0.018–4.607
*GASBOR* (*ab initio* chain-like modelling)	
χ^2^ value	1.7
*s* range (nm^−1^)	0.143–4.607
Symmetry	*P*2
*SREFLEX* (modelling of flexibility based on crystal structure)	
χ^2^ value	5.7
*s* range (nm^−1^)	0.018–4.607
Software	
Data processing and basic analyses	*SASFLOW* (Franke *et al.*, 2012 ▸; Blanchet *et al.*, 2015 ▸) and *PRIMUSqt* (Petoukhov *et al.*, 2012 ▸)
Distance distribution analysis	*GNOM* (Svergun, 1992 ▸) through *PRIMUSqt* (Petoukhov *et al.*, 2012 ▸)
*Ab initio* analysis	*GASBOR* (Svergun *et al.*, 2001 ▸) *via ATSAS* online (https://www.embl-hamburg.de/biosaxs/atsas-online/)
Conformational analysis	*SREFLEX* (Panjkovich & Svergun, 2016 ▸) *via ATSAS* online (https://www.embl-hamburg.de/biosaxs/atsas-online/)
Comparison to crystal structure	*CRYSOL* (Svergun *et al.*, 1995 ▸)
Graphics representation	*PyMOL* (http://www.pymol.org)
Extinction coefficient estimate	*ProtParam* (Gasteiger *et al.*, 2005 ▸)

**Table 4:** Structural homologues of *Mm*GADL1 The homologues were detected by an *SSM* analysis using *PDBeFold*.

Protein	Organism	PDB entry	Reference	Chain	*Q*-score	R.m.s.d. (Å)	Sequence identity (%)	UniProtKB entry	Aligned residues
*Mm*GADL1	*Mus musculus*	6enz	This study	*A*	—	—	—	E9QP13	1–502
*Hs*CSAD	*Homo sapiens*	2jis	Unpublished work	*B*	0.586	0.85	62.0	Q9Y600	1–493
*Hs*GAD65	*Homo sapiens*	2okk	Fenalti *et al.* (2007 ▸)	*A*	0.565	1.06	49.4	Q05329	88–584
*Hs*GAD67	*Homo sapiens*	2okj	Fenalti *et al.* (2007 ▸)	*B*	0.532	1.15	50.9	Q9925	93–594
*St*PDD	*Sphaerobacter thermophilus*	4rit	Unpublished work	*B*	0.461	1.96	28.9	D1C7D8	1–483
RUMGNA_01526	*Ruminococcus gnavus*	4obu	Williams *et al.* (2014 ▸)	*H*	0.492	1.79	25.6	A7B1V0	1–490
*Vp*GAD	*Vibrio parahaemolyticus*	2qma	Unpublished work	*B*	0.442	2.16	25.8	Q87NC6	464–957
*Hs*HDC	*Homo sapiens*	4e1o	Komori *et al.* (2012 ▸)	*C*	0.425	2.13	26.1	P19113	2–477
*Hs*DDC	*Homo sapiens*	3rbl	Giardina *et al.* (2011 ▸)	*A*	0.397	2.61	23.3	P20711	1–480
