# Supplementary material for: Structure of the mouse acidic amino acid decarboxylase GADL1
Source: Acta Crystallogr F Struct Biol Commun. 2018 Jan 1;74(Pt 1):65–73. doi: 10.1107/S2053230X17017848 (PMC5947694; doi:10.1107/S2053230X17017848)
Supplement: Supplementary file 2 [file f-74-00065-sup2.pdf]

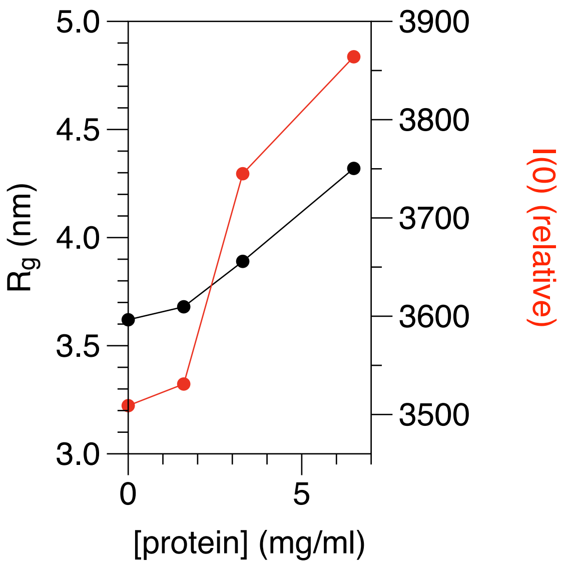

Supplementary Figure 1. Rg and I(0) as a function of protein concentration in the SAXS experiment. The dataset extrapolated to zero concentration and used in all the analyses is included.
